# Data Analysis tools for the Compact X-ray Light Source and Compact X-ray Free Electron Laser facilities at Arizona State University

**DOI:** 10.1063/4.0000866

**Published:** 2025-10-27

**Authors:** Sabine Botha, Annelise Velarde, Gihan Ketawala, Eric Everett, Roberto Alvarez, Thomas D Grant, Richard A Kirian

**Affiliations:** 1 Arizona State University, Tempe, AZ, USA; 2 University of Buffalo, Buffalo, NY, USA

## Abstract

The Compact X-ray Light Source (Figure 1) and Compact X-ray Free Electron Laser [1], [2] that are currently undergoing commissioning and construction, respectively, at Arizona State University will be the first of their kind with applications in structural biology, medical imaging, atomic, molecular and optical physics as well as studying condensed matter and quantum materials.

The unique characteristics of the ultrashort X-ray pulses produced by both sources at kilohertz repetition rates necessitate novel and new data analysis algorithms and tools as well as supportive cyber infrastructure at ASU. Here we will report on the current state of the data analysis support and tools currently under development at CXLS/CXFEL for studying biological macromolecules at these sources for both online (real- time) and offline analysis. These include an AI/ML-based crystal hitfinder, integration of an AI/ML-based image sorter [3], Small Angle X-ray Scattering analysis tools, calibration pipelines etc.

